# Segmentation of lung lobes and lesions in chest CT for the classification of COVID-19 severity

**DOI:** 10.1038/s41598-023-47743-z

**Published:** 2023-11-28

**Authors:** Prachaya Khomduean, Pongpat Phuaudomcharoen, Totsaporn Boonchu, Unchalisa Taetragool, Kamonwan Chamchoy, Nat Wimolsiri, Tanadul Jarrusrojwuttikul, Ammarut Chuajak, Udomchai Techavipoo, Numfon Tweeatsani

**Affiliations:** 1 Centre of Learning and Research in Celebration of HRH Princess Chulabhorn’s 60th Birthday Anniversary, Chulabhorn Royal Academy, Bangkok, Thailand; 2 Chulabhorn Hospital, Chulabhorn Royal Academy, Bangkok, Thailand; 3 Department of Computer Engineering, Faculty of Engineering, King Mongkut’s University of Technology Thonburi, Bangkok, Thailand; 4 Princess Srisavangavadhana College of Medicine, Chulabhorn Royal Academy, Bangkok, Thailand; 5 Queen Savang Vadhana Memorial Hospital, Chonburi, Thailand; 6 Faculty of Health Science Technology, HRH Princess Chulabhorn College of Medical Science, Chulabhorn Royal Academy, Bangkok, Thailand

**Keywords:** Computational biology and bioinformatics, Image processing, Machine learning, Medical imaging, Computed tomography

## Abstract

To precisely determine the severity of COVID-19-related pneumonia, computed tomography (CT) is an imaging modality beneficial for patient monitoring and therapy planning. Thus, we aimed to develop a deep learning-based image segmentation model to automatically assess lung lesions related to COVID-19 infection and calculate the total severity score (TSS). The entire dataset consisted of 124 COVID-19 patients acquired from Chulabhorn Hospital, divided into 28 cases without lung lesions and 96 cases with lung lesions categorized severity by radiologists regarding TSS. The model used a 3D-UNet along with DenseNet and ResNet models that had already been trained to separate the lobes of the lungs and figure out the percentage of lung involvement due to COVID-19 infection. It also used the Dice similarity coefficient (DSC) to measure TSS. Our final model, consisting of 3D-UNet integrated with DenseNet169, achieved segmentation of lung lobes and lesions with the Dice similarity coefficients of 91.52% and 76.89%, respectively. The calculated TSS values were similar to those evaluated by radiologists, with an R2 of 0.842. The correlation between the ground-truth TSS and model prediction was greater than that of the radiologist, which was 0.890 and 0.709, respectively.

## Introduction

The rapid pandemic-level outbreak of coronavirus disease 2019 (COVID-19) has caused a wide range and degree of illnesses, predominated by respiratory tract infection^1–4^. Although most infected patients show asymptomatic or mild clinical manifestations, further investigation beyond real-time reverse transcriptase polymerase chain reaction (RT-PCR) or rapid COVID-19 tests such as chest radiographs is routinely indicated in worsening cases that require hospitalization^5,6^. Characteristic findings in chest radiographs of COVID-19 related pneumonia are bilateral patchy and/or confluent and bandlike ground-glass opacity or consolidation in a peripheral and mid-to-lower lung zone distribution. By contrast, several studies have found almost one-half of normal chest radiographs at initial presentation disagree with clinical symptoms^7–10^.

Because of its higher sensitivity, specificity, and speed, chest computed tomography (CT) has become more useful than RT-PCR in early detection, to obtain more information about chest pathology, and to evaluate the severity of lung involvement. Moreover, it can assist triage, especially when hospitalization is required but there is a shortage of healthcare personnel, inpatient beds, and medical equipment, and it may be useful as a standard modality for the rapid diagnosis of COVID-19- related pneumonia^11–15^. The chest CT findings are peripheral, bilateral, ground-glass opacity (GGO) with some round shapes with or without consolidation or intralobular lines, a reverse halo sign, or other findings of organizing pneumonia^16–19^.

The total severity score (TSS) has been proposed by Chung et al.^20^. It is calculated from the summation of lesion scores in five lung lobes and is used to categorize the severity of lung involvement and help determine the proper therapeutic management and prognosis^21,22^. TSS reflects the clinical classification of COVID-19^22^. It has also been shown to provide high specificity in the detection of severe cases and high inter-observer reliability with a short interpretation time compared to other severity scoring system^23^. It has been used in many studies, such as the comparison of patients with and without vaccination^24^, and the viral load factor for hospitalization and mortality of patients^25^.

To reduce the amount of time required for interpretation and increase the accuracy of lesion detection, deep learning has been used to efficiently analyze medical images by performing tasks such as semantic segmentation. Deep learning was also used in the automated assessment of CT severity scores in COVID-19 patients. Lessmann et al.^26^ applied deep-learning algorithms that automatically segment the five pulmonary lobes and abnormalities and then predict the severity scores for patients suspected of having COVID-19. The results showed good agreement with the results from independent observers. Chaganti et al.^27^ automatically computed the percentage of opacity and lung severity score by applying deep reinforcement learning for lung lobe segmentations and using the U-Net model for a semantic segmentation of GGO and consolidations. The results correlated well with the ground truth.

The U-Net model is a convolutional neural network-based model that was originally used for the semantic segmentation of biomedical images and is now one of the most utilized image segmentation techniques. The model structure is U-shaped and consists of two parts: a contracting path (encoder) and an expanding path (decoder)^28^. Subsequently, a U-Net model was created to support three-dimensional (3D) matrices and is called 3D-Unet^29^. The 3D-UNet model was used to develop a more efficient 3D imaging model for the segmentation of lesions and lung tissue^30,31^. Cropping the lung area before lesion segmentation can improve accuracy^32^. Enshaei et al.^33^ developed a model for predicting the lesion area of COVID-19 patients from CT-scan images, using a model to predict the lung area before the lesion regions were considered. This method enables the lesion model to predict lesions more accurately. In another study, a deep learning model was applied to lung lobe segmentation. The model is capable of accurately segmenting each lung lobe from lung CT scans^34^. It is also utilized in lung lobe segmentation analysis for lung segmentation research to improve segmentation accuracy in multiple diseases such as chronic obstructive pulmonary disease (COPD), lung cancer, and COVID-19-related pneumonia^35^.

Many studies have used deep learning models for computer-aided diagnostics to determine the intensity of infections. For instance, Aswathy A. L. and Vinod Chandra S. S.^36^ employed 3D-UNet models to effectively segment the lung parenchyma and infected regions in lung CT scans. Additionally, a previous study demonstrated that the effectiveness of these models for medical image segmentation can improve sensitivity performance^37^. In another study, the U-Net model combined with the dense convolutional network (DenseNet) was effectively employed to develop a program for classifying the severity of lung CT in COVID-19 by analyzing the lesion area and comparing it with the lung area in lung CT scan images^38^. They calculated the percentage of infection (PI) using a U-Net model combined with pre-trained models such as residual neural networks (ResNet) and DenseNet. ResNet was first presented by He et al.^39^ to solve the vanishing gradient problem of deeper networks by adding feedforward links across some layers, resulting in residual optimization of those layers. DenseNet was first presented by Huang et al.^40^ to learn more features by using deeper convolutional layers with many feedforwards linking across layers. For this reason, this knowledge can be applied to lung lobe segmentation and lesion segmentation in CT scan images.

In this study, deep learning semantic segmentation was used for the lung severity scoring of the COVID-19 infection. The proposed method utilized a combination of 3D-UNet models integrated with pre-trained models, DenseNet and ResNet, to compute the PI from the lung lobe and lesion segmentation results and estimate the TSS automatically. The aim was to alleviate the radiologist's workload and time spent on imaging diagnostics, as well as improve reporting accuracy.

## Materials and methods

### Datasets

Due to its retrospective nature, informed consent was waived, and all data were anonymized. This project was approved by the human research ethics committee of the Chulabhorn Research Institute (research project code 167/2564) and complied with the Declaration of Helsinki. These COVID-19 patients were confirmed by RT-PCR acquired from Chulabhorn Hospital who underwent non-contrast enhanced axial chest CT as a part of routine clinical care throughout the pandemic.

In this study, we randomly selected 124 cases from the database. The selection contained 28 cases without lung lesions and 96 cases with lung lesions. According to TSS, experienced radiologists classified the cases with lung lesions as mild, moderate, and severe. We divided the selection into 3 groups, i.e., training set, test set 1, and test set 2. The training set was used in model training and validation for lung segmentation and lesion segmentation; test set 1 was for segmentation performance evaluation; and test set 2 was for TSS prediction evaluation. We also randomly selected these cases for each group. In addition, for the training set and test set 1, the numbers of cases across different severity types were set to be equal to prevent class imbalance in the training set (the class imbalance causing a potential bias in the trained model) and for a fair comparison in test set 1. The number of CT slices in these cases ranged from 92 to 208. This information was described in Table 1.

**Table 1:** Summary of axial lung CT scan datasets.

Case type	Training set			Test set 1			Test set 2		
	No. of cases	Total slices	Avg ± std per case	No. of cases	Total slices	Avg ± std per case	No. of cases	Total slices	Avg ± std per case
No lesions	8	985	123 ± 22	5	705	141 ± 39	15	1953	130 ± 27
Mild	8	907	113 ± 12	5	707	141 ± 48	16	2119	132 ± 27
Moderate	8	941	118 ± 13	5	575	115 ± 20	27	3179	118 ± 27
Severe	8	919	115 ± 23	5	597	119 ± 36	14	1469	105 ± 12
Total	32	3752	117 ± 18	20	2584	129 ± 36	72	8720	121 ± 26

### Data preprocessing

The lung CT data were saved in JPEG format with a resolution of 512 × 512 pixels and labeled by a program called LabelME^41^ (version 4.5.12). The resulting labels were in JavaScript Object Notation (JSON) format. All labeled data were validated by four radiologists and then converted into matrices for model training and evaluation.

**Figure 1:**
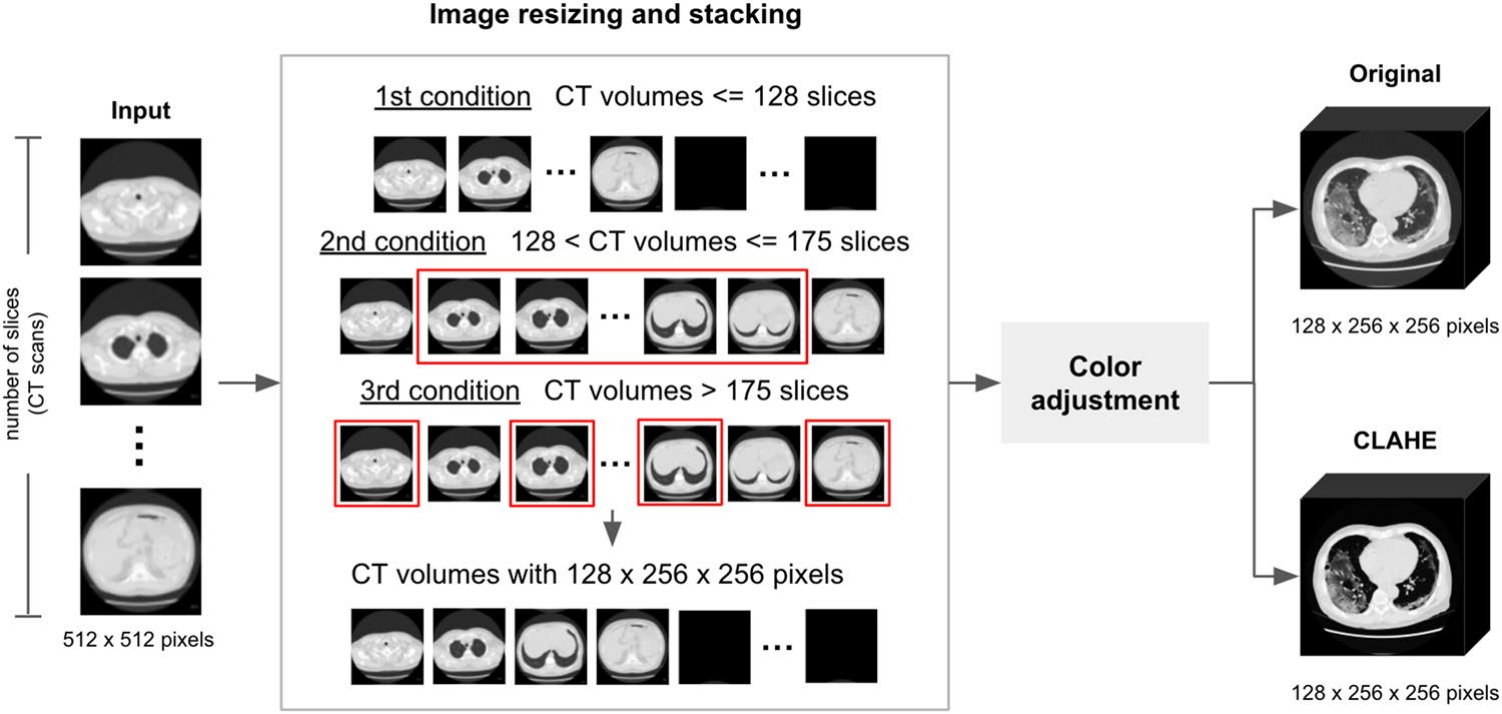
Overall pipeline of the data preprocessing.

In the data preprocessing phase, CT scan images (JPEG format) and labeled data (JSON format) were resized using the “cv2.resize” function from 512 × 512 pixels to 256 × 256 pixels to minimize the required memory resources (RAM). The interpolation parameter was set to “INTER_AREA” for the CT scan images and “INTER_NEAREST" for the label data because this solution prevented any alteration of the values specified in each pixel. In addition, our model input shape was fixed at a size of 128 × 256 × 256. The CT volumes were adjusted to 128 images per patient according to the following three conditions (Fig. 1).

- The first condition, if the CT volume comprised 128 or fewer images, a 256 × 256 zero-padding matrix was added to increase the volume to 128 images.
- The second condition, if the CT volume had between 129 and 175 CT images, 128 images from the CT volume's middle range were selected to train the model because both the lung parenchyma and lesions appear in this range.
- The third condition, if the CT volume contained more than 175 CT images, we skipped the CT slice by selecting only odd-numbered images and adding a 256 × 256 zero-padding matrix to reach a total of 128 images.

A color adjustment method was applied to improve image contrast by using the contrast-limited adaptive histogram equalization (CLAHE) technique^42^, which is available in the OpenCV library^43^. The CLAHE parameters were set to a clipLimit of 3 and a tileGridSize of (8, 8). Models were trained/tested in two experiments: the first with original images (no color adjustment) and the second with CLAHE-adjusted images.

### Imaging protocol

A 256-slice dual-energy CT scanner (Revolution CT with Gemstone Spectral Imaging (GSI) Xtream, GE Healthcare) at Chulabhorn Hospital was used in this study. An axial chest CT scan without contrast agent was applied. The protocol started with a scout view from lung apices to lung bases in anterior–posterior (AP) and lateral views, and followed by an axial chest scan covering lung apices through bases from inferior to superior. The parameters were quiet breath inspiration, 1.25 mm thickness, 0.28 s/rotation, 0.992 pitch, GSI calculated kVp, 190 mA, lung window of (1550, − 700), soft tissue window of (400, 40), and postprocessing multiplanar reconstruction. The scan time was less than 1.6 s.

### Model training

Two models were used in this study: (1) a lung lobe segmentation model and (2) a lesion segmentation model. Training set: 32 cases were split into 24 cases (75%) for model training and 8 cases (25%) for validation, where the dataset was divided equally at each severity type to prevent overfitting. According to related studies, a model that combines a 3D-UNet structure with a DenseNet or ResNet is effective in segmenting parts of the image precisely. Therefore, pre-trained DenseNet121, DenseNet169, DenseNet201, ResNet18, ResNet34, ResNet50, ResNet101, and ResNet152 models were obtained through a segmentation-models-3D package from Solovyev et al.^44^.

*Lung lobe segmentation.* A multiclass semantic segmentation model was used to segment the five lung lobes. Annotated labels consisted of six categories: 0, 1, 2, 3, 4, and 5, which indicate the background, right upper lobe (RUL), right lower lobe (RLL), right middle lobe (RML), left upper lobe (LUL), and left lower lobe (LLL), respectively.

*Lesion segmentation.* The lesion model was developed from a binary semantic segmentation model that outputs the value 1 for lesion areas and 0 for background areas. Images without extrapulmonary regions are preferred for lesion model training. The dataset used for model training was preprocessed as described in the data preparation section.

**Figure 2:**
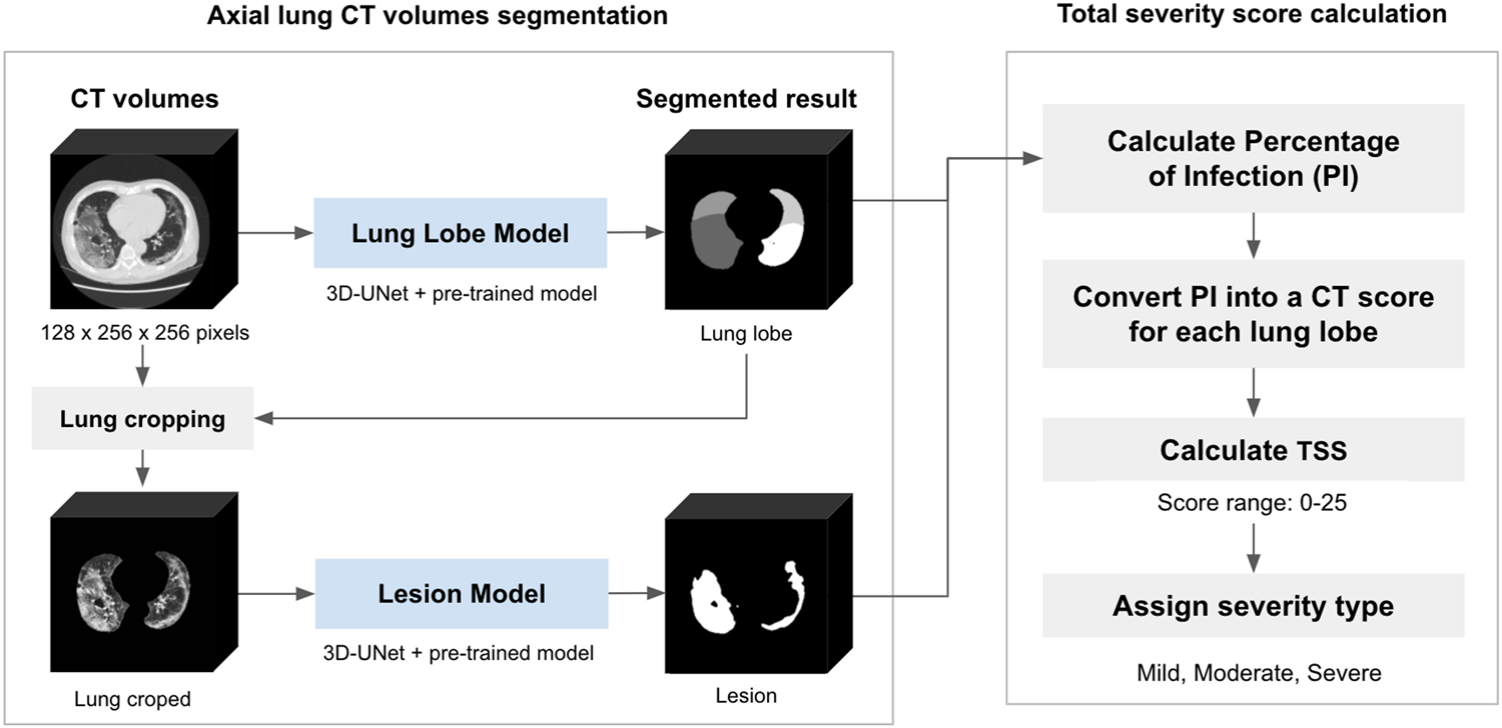
Segmentation Model Workflow and Total Severity Score Calculation Protocol for Lung CT Scans.

In the model training process, lung lobe and lesions segmentation models were trained on servers equipped with an Intel(R) Xeon(R) Gold 6126 CPU at 2.60 GHz, 40 GB of RAM, and an NVIDIA Tesla V100 SXM2 GPU. Figure 2 shows the overall workflow. The model’s output is the predicted class for each pixel, which is then used to compute the percentage area of lesions in each lung lobe for the CT score. This score is then used to calculate the TSS value for diagnosing the severity of the current pathology. For both models, Adam optimization was used, the loss function was a hybrid loss function (focal loss + Dice loss), the learning rate was set to 0.0001, a regularizer that applies L2 regularization was used with a value of 0.01, the batch size was set to 1, and the maximum number of epochs was 200. The lesion model activation function was set to sigmoid with a dropout rate of 0.4, whereas the pulmonary lobe model activation function was set to SoftMax with a dropout rate of 0.2. The hybrid loss technique^45^, which combined focal loss and Dice loss, was used to improve model performance.

### PI

The PI in each lung lobe was calculated by dividing the number of predicted lesion pixels by the total number of lung lobe pixels in the CT volume. The predicted lesion pixels were obtained from the output of the lesion segmentation model, whereas the predicted lung lobe pixels were derived from the output of the lung lobe segmentation model, in which the value of each pixel identifies the lobar type in the lung CT image. Therefore, the PI was calculated by performing the following equation.

$$Percentage\, of{\, Infection}_{lobe}= \frac{Lesion\, Area\, (pixels)}{Lung \,Lobe\, Area\, (pixels)}\times 100$$

### TSS

The TSS proposed by Chung et al.^20^ was calculated from the sum of the five-lobe CT score, which was calculated from PI based on the criteria listed in Table 2. The severity of COVID-19 patients can be classified from the TSS value based on the severity criteria in Table 3 and the following equation:

**Table 2:** CT-score criteria based on the percentage of infection (PI) for the diagnosis of pneumonia in COVID-19 patients proposed by Chung et al.^20^.

PI	Score
No lesion	0
< 5%	1
6–25%	2
26–50%	3
51–75%	4
> 75%	5

**Table 3:** Severity type classification based on the total severity score (TSS).

TSS	Severity type
≤ 7	Mild
8–17	Moderate
≥ 18	Severe

$$TSS =CT \,Scor{e}_{RUL}+CT \,Scor{e}_{RML}+CT \,Scor{e}_{RLL}+CT\, Scor{e}_{LUL}+CT\, Scor{e}_{LLL}$$

### Dice similarity coefficient

The most commonly utilized measurement to evaluate image segmentation is the Dice similarity coefficient (DSC). The DSC calculated the relative overlap between the predicted area and ground truth, and it was used to choose the most appropriate model. The DSC was defined as follows:

$$DSC = \frac{2TP}{2TP + FP + FN}$$

where the term “true positive” (TP) refers to an outcome such that the model correctly predicts the positive class, “false positive” (FP) is an outcome such that the model incorrectly predicts the positive class, and “false negative” (FN) is an outcome such that the model incorrectly predicts the negative class.

### Hausdorff distance

Hausdorff distance was proposed by Felix Hausdorff in 1914^46,47^. The measure was applied to evaluate the model’s performance by measuring the distance between two images in pixels. The distance is defined as

$$H\left(A,B\right)=\mathrm{max}\left(h\left(A,B\right), h\left(B,A\right)\right)$$

$$h\left(A,B\right)={\mathrm{max}}_{\mathit{a\epsilon A}}{\mathrm{min}}_{b\in B}||a-b||,$$

where A is a set containing *p* points (pixels on image A): $\{{a}_{1},{a}_{2},\dots ,{a}_{p}\}$ and B is a set containing *q* points (pixels on image B): $\{{b}_{1},{b}_{2},\dots ,{b}_{q}\}$. For implementation, we applied the function implemented in SciPy package^48^. Since the images used in this research were 256 × 256 pixels, the Hausdorff distance range was $[0, 256\sqrt{2}]$.

## Results

### Lung lobe segmentation

The testing of lung lobe segmentation models was divided into two sections, one for the models trained on the original images and one for the models trained on the lung CT images processed by the CLAHE technique. The model's test results for test set 1 were given in Supplementary Tables S1 and S2. The 3D-UNet + DenseNet169 model trained with the original CT data was found to obtain the best lung lobe segmentation with a DSC of 91.52% and an average Hausdorff distance of 12.9 pixels.

**Figure 3:**
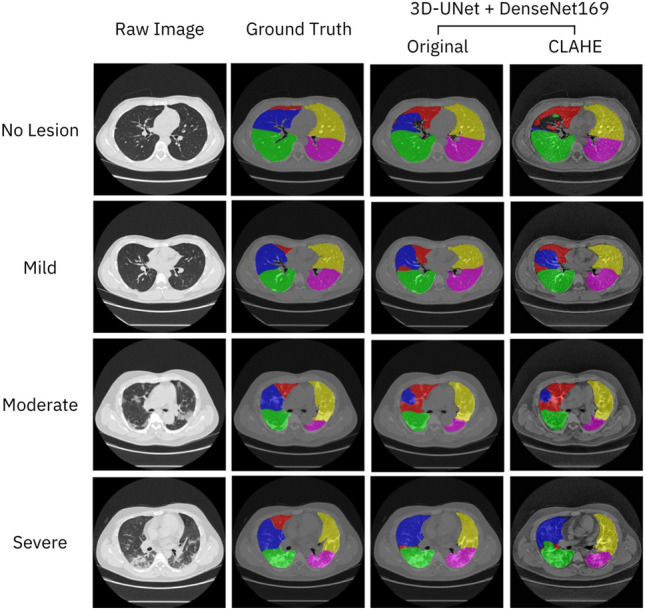
Examples lung lobe segmentation results for each severity level obtained by the 3D-UNet + the DenseNet169 model trained on the original images and images processed by CLAHE. Each lobe of the lung, right upper lobe (RUL), right lower lobe (RLL), right middle lobe (RML), left upper lobe (LUL), and left lower lobe (LLL), is indicated by the colors red, blue, green, yellow, and pink, respectively.

For lung lobe segmentation, the results indicated that the model trained on the original images outperformed the model trained on the CLAHE-adjusted images, as indicated in Table 4. Figure 3 presented the image segmentation results for both the original and CLAHE-adjusted images. In the case of the middle lobe (blue) of the no-lesion group, the model trained on the original images performed better than the model trained on CLAHE-adjusted images, which erroneously labeled as the right upper lung (red) and the background area.

**Table 4:** Lung lobe and Lesion segmentation results of the 3D-UNet + DenseNet169 model with test set 1 (n = 20) showing Dice similarity coefficient (DSC), Hausdorff distance (HD), and their ± standard deviations.

	Non-CLAHE		CLAHE	
	DSC (%)	HD (pixels)	DSC (%)	HD (pixels)
Lung lobe segmentation				
(Divided into lobes)				
RUL	89.52 ± 4.58	13.38 ± 7.23	89.95 ± 4.74	13.55 ± 7.40
RML	90.56 ± 4.22	16.46 ± 8.90	88.91 ± 5.14	17.45 ± 9.97
RLL	91.29 ± 4.20	9.41 ± 5.37	91.21 ± 4.64	10.91 ± 5.71
LUL	92.72 ± 3.16	15.91 ± 5.29	92.26 ± 3.40	17.51 ± 7.29
LLL	93.53 ± 2.33	9.36 ± 6.80	92.32 ± 4.67	9.28 ± 4.66
(Divided into severity types)				
No lesion	91.86 ± 0.52	12.94 ± 2.35	91.42 ± 2.47	14.78 ± 6.54
Mild	90.36 ± 1.35	15.11 ± 3.45	90.03 ± 2.92	15.31 ± 5.10
Moderate	92.70 ± 2.39	12.09 ± 5.23	92.47 ± 3.14	11.91 ± 4.98
Severe	91.16 ± 4.09	11.48 ± 5.96	89.80 ± 5.60	12.95 ± 6.33
Overall	**91.52 ± 2.44**	**12.90 ± 4.35**	**90.93 ± 3.61**	**13.74 ± 5.49**
Lesion segmentation				
No lesion*	93.02 ± 8.24	6.53 ± 14.59	90.3 ± 14.49	11.75 ± 26.28
Mild	75.92 ± 13.17	47.80 ± 34.19	77.78 ± 6.92	40.19 ± 29.33
Moderate	65.18 ± 10.32	57.88 ± 11.66	67.85 ± 8.00	55.08 ± 14.10
Severe	67.77 ± 12.58	42.98 ± 5.99	71.62 ± 16.87	44.25 ± 24.69
Overall	**75.47 ± 15.2**	**38.80 ± 26.89**	**76.89 ± 14.28**	**37.82 ± 27.68**

The overall performance values are in bold.

*Predicting lung area without lesions instead of predicting lesion area.

Segmentation results for each severity level indicated that the mild group underperformed the other groups for the model trained on the original images. The DSC values for each lobe segment were presented in Table 4 and Supplementary Tables S3, revealing that the left lobe (upper and lower) segmentation was more accurate than the right lobe segmentation. However, the right and left lower lobes (RLL and LLL) exhibited lower boundary distances, with HD values of 9.41 and 9.36 pixels, respectively.

### Lesion segmentation

The results of the lesion model on test set 1 were divided into two sections, one for the original images and one for the images processed using the CLAHE technique (Supplementary Tables S4 and S5). The models trained with the CT images processed by CLAHE were found to be the most effective for lesion segmentation, with the 3D-UNet + DenseNet169 model obtaining a DSC of 76.89% and an average Hausdorff distance of 37.82 pixels.

**Figure 4:**
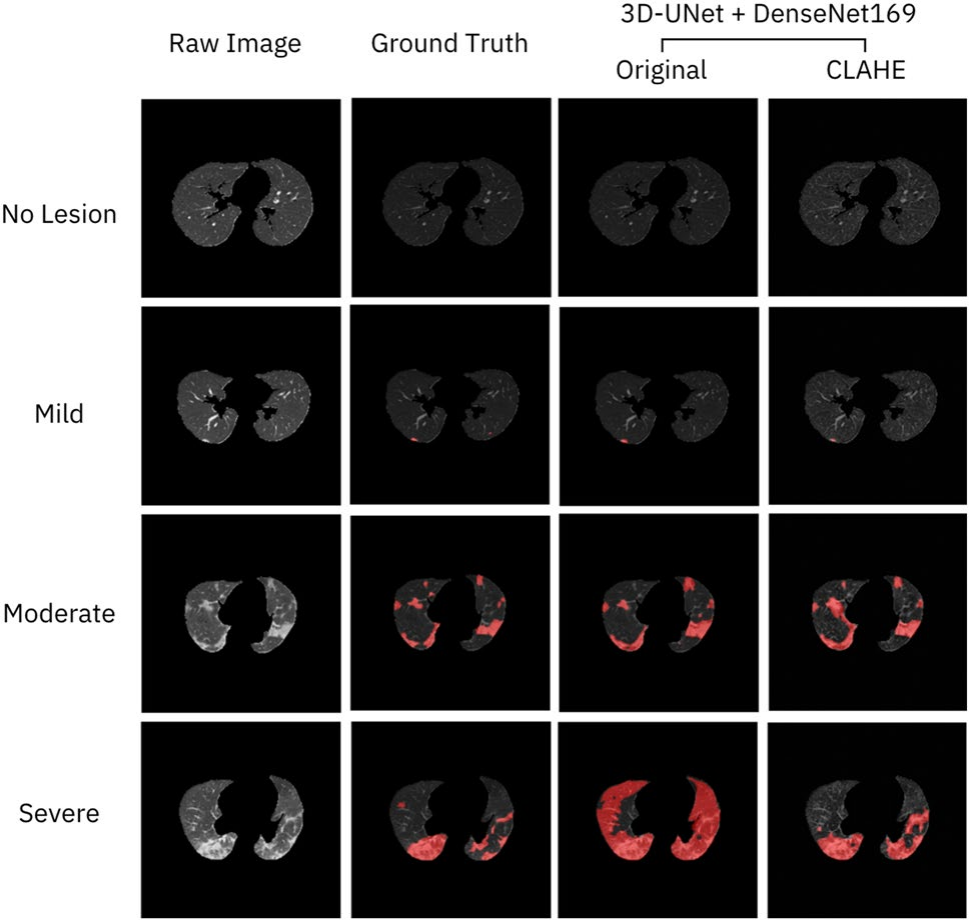
Example of segmentation results obtained by models trained on the original images and images processed by the contrast-limited adaptive histogram equalization (CLAHE) at each level of severity. The red pixels indicate the lesion areas.

According to the test results, the model trained on the CLAHE-adjusted images performed better than the model trained on the original images. The best model (3D-UNet + DenseNet169) was evaluated on test set 1, and the results were shown in Table 4 and Fig. 4. These results implied that lesion models often accurately predicted lesions when images were completely free of lesions (no-lesion images) and obvious lesions like consolidation, while faint lesions such as GGO were often less accurate in prediction.

### TSS regression analysis

**Figure 5:**
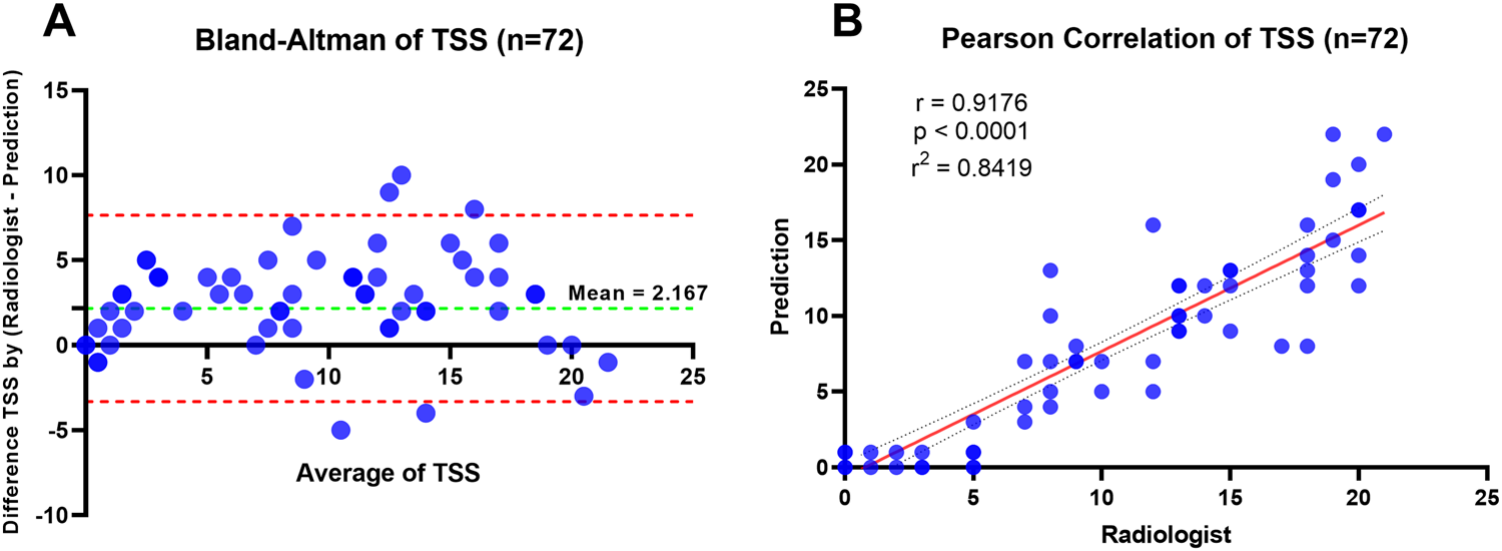
Distribution of TSS values obtained by our model prediction and a radiologist for test set 2 (n = 72). (**A**) Bland–Altman plot illustrating the comparison of TSS values between the radiologist and prediction. (**B**) Regression plot depicting the correlation between TSS calculated from the radiologist and prediction.

The best-performing lung and lesion models with the highest DSC were applied to perform image segmentation for 72 cases (test set 2). The segmentation results were used to calculate the PI in each lung lobe, which was then used to compute the TSS. To analyze the trends and correlation of the TSS results, we compared the radiologist measurements with TSS values calculated based on our approach using Pearson correlation coefficients (r) and R^2^ values. Figure 5 presented the results of the analysis of the TSS values obtained by models and those obtained by radiologists. According to the appropriate statistical analysis, the Pearson correlation coefficient of 0.918 indicated that the model-based predictions and the radiologist's diagnosis were positively correlated with an R^2^ of 0.842.

To compare the outcomes of TSS that our approach predicted and those that radiologists diagnosed, we used the Bland–Altman plot. The x-axis represented the range of the TSS score and the y-axis represents the difference in the score of TSS between the two methods. It was found that the 95% limits of agreement, or the range of values within -3.318 to 7.652. The mean difference between the two methods was 2.167, which means that the radiologists diagnosed more than TSS predicted on average by 2.167, as shown in Fig. 5A.

**Figure 6:**
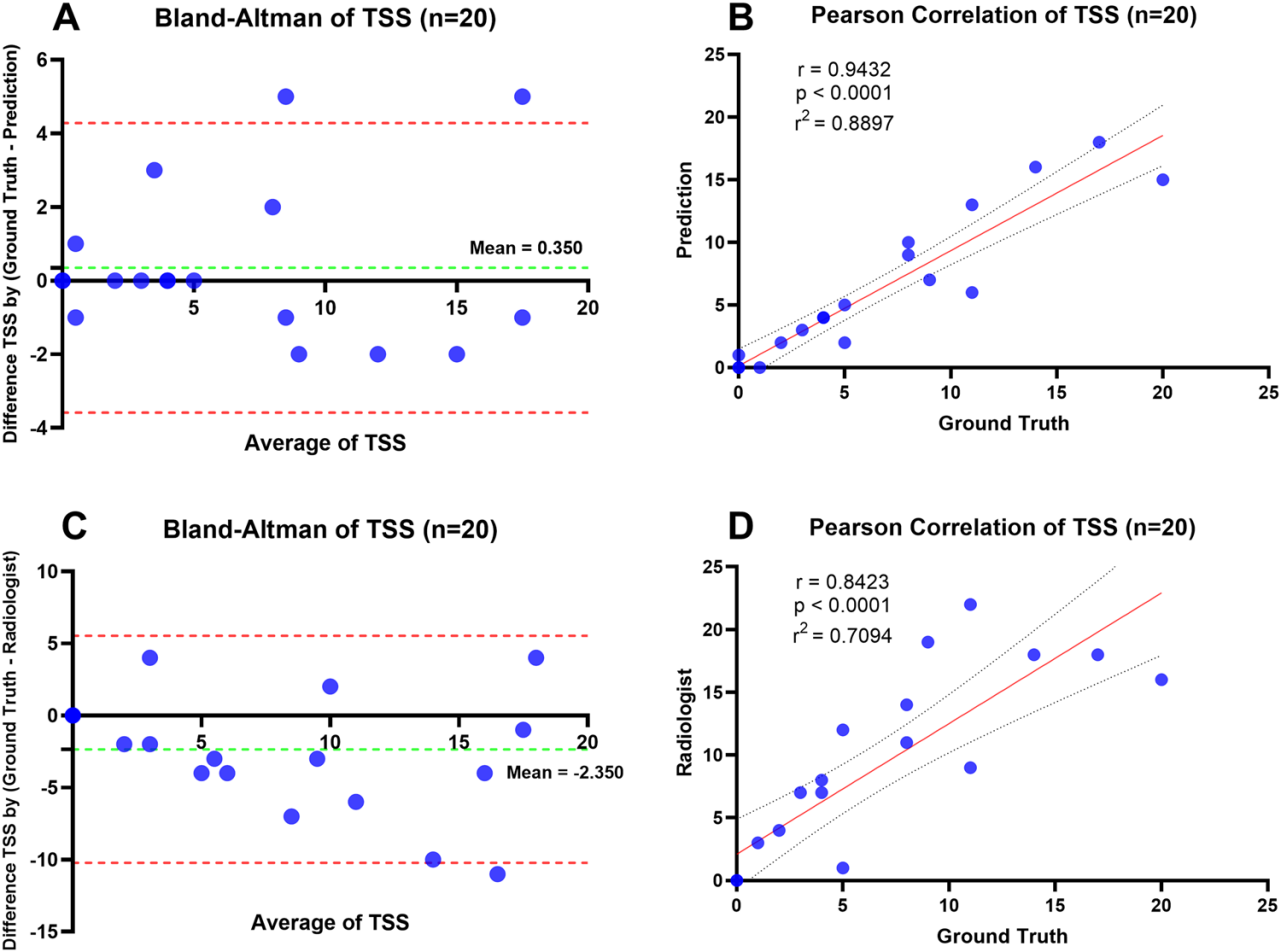
Distribution of TSS values for method comparison with ground truth using Test Set 1. (**A**) Bland–Altman plot illustrating the comparison of TSS values between the ground truth and prediction. (**B**) Regression plot depicting the correlation between TSS calculated from the ground truth and TSS predicted. (**C**) Bland–Altman plot demonstrating the comparison of TSS values between the ground truth and radiologist. (**D**) Regression plot analysis showcasing the correlation between TSS values calculated from the ground truth and those provided by the radiologist.

Additionally, three techniques for measuring the TSS value were compared: using the label mask of test set 1 as the ground truth, the radiologist's diagnostic TSS (Radiologist), and the model-based TSS (Prediction). TSS (Radiologist) indicated the radiologist determined the TSS value. TSS (GT) indicated the TSS value obtained by using the ground truth for the calculation. TSS (prediction) was the TSS result obtained using the model predictions for the computation. The outcomes of the investigation were presented in Fig. 6. The TSS (Prediction) values were correlated with TSS (GT) values, resulting in an R^2^ of 0.890. Furthermore, the R^2^ of 0.709 from the comparison between TSS (Ground Truth) and TSS (Radiologist) indicated a higher correlation for the model compared to a human.

### Ethical approval

Ethical approval was obtained from the human research ethics committee of the Chulabhorn Research Institute (research project code 167/2564).

### Informed consent

Informed consent was waived by the human research ethics committee of the Chulabhorn Research Institute due to retrospective study and all data were anonymized.

## Discussion

We compared the proposed lung lobe segmentation model's performance with the performance of models in other related studies. Tang et al.^45^ reported a DSC of 91.48% on the LUNA16 dataset and 94.17% on the Tianchi dataset, indicating that our model underperformed theirs. However, Tang et al. employed CT datasets of lungs without lesions for their test datasets, and the models were trained on 40 cases, whereas our model utilized the no-lesion training dataset, which consisted of eight cases. The performance of our model (3D-UNet + DenseNet169) was equivalent to that of Tang et al., with a DSC of 91.86% in the no-lesions group (Table 4). This demonstrates that our approach can be trained using a small dataset. Furthermore, a comparison with a lung segmentation algorithm based on the lung fissure surface yielded a DSC of 84.00%^49^. This indicated that a deep learning approach could efficiently enhance the performance of lung lobe segmentation, particularly due to the continuous training of 3D-Unet on a complete set of data by inputting entire CT slices per case. As a result, the 3D-Unet model was suitable for precise lung lobe segmentation in continuous CT images.

For the lesion segmentation studies, Xiao et al.^30^ reported a 3D-UNet model with a DSC of 89.12%, and Qiblawey et al.^38^ reported a DSC of 94.13% for the Feature pyramid network (FPN) combined with the DenseNet201 model. Both models outperformed ours because training a model required a considerable variety of data. For example, Qiblawey et al.^38^ used 15,698 images for training, whereas our model training used a limited number of 3752 images. Despite the smaller dataset, our lesion model's results demonstrated that it was capable of accurately predicting lesions in the no-lesion and obvious lesions, despite the inaccuracies in lesion segmentation for faint lesions. Many approaches that should improve the performance of lesion segmentation include increasing the amount of training data, applying data augmentation to increase the variety of lesions, enhancing the contrast rate, and utilizing the recent developments in state-of-the-art segmentation models, e.g. PaddleSeg^50^, to achieve improvements in accurately segmenting lesions from medical imaging data. These approaches would be potential solutions included in our future work.

Image enhancement using CLAHE could not be suitable for the lung lobe segmentation model. The results showed that the lung model trained on the original image accurately segmented better than the model trained on CLAHE images. It might be possible that CLAHE locally emphasizes many details on the image, and thus making the lung fissures more difficult to detect. In contrast, for the lesion segmentation model, using CLAHE provided a small improvement. Other enhancement methods such as the Balance Contrast Enhancement Technique (BCET)^51^ could be a potential solution for enhancing contrast quality and preserving the histogram pattern of the image. As shown in a previous study focusing on COVID-19 classification using lung X-ray images, BCET outperformed CLAHE^52^.

Additionally, the TSS regression analysis showed that our model can be utilized to segment lung CT scans effectively. An analysis of the radiologist's diagnostic TSS and model-based TSS results revealed that our method produced observation results that were more accurate with respect to the ground truth than human observation. This demonstrates that the measurement software could be used to eliminate human error in the estimation of infection rates in the lung area. Furthermore, our approach could perform lesion area segmentation and calculate the infection rate automatically. This could dramatically reduce the radiologist’s workload and enhance the efficiency of diagnosing COVID-19 severity levels.

TSS is a rapid and objective assessment method for radiologists that provides information in each lobe and is more feasible for manual evaluation. However, it grades each lung lobe equally significantly, without concerning about their volume differences. In contrast, PI calculates the entire affected volume, resulting in a more accurate evaluation of infected lung volume. Nevertheless, some remote hospitals lack an automated program, and TSS appears to be an effective evaluation instrument.

## Conclusion

Constructing a model for the automatic segmentation and scoring of COVID-19 infection in chest CT was accomplished through the application of deep learning techniques. According to the findings, the combination of 3D-UNet and DenseNet169 achieved the highest level of performance when it came to the segmentation of lung lobes and lesions. The projected severity score had a strong correlation with the visual assessments made by radiologists. This accurate model provided a dependable method for quantifying the extent of lung involvement. The proposed model was helpful in determining the extent of the lower respiratory tract infection and monitoring the disease in COVID-19 patients.

### Supplementary Information

Supplementary Information.

## Data Availability

The datasets used and/or analyzed during the current study available from the corresponding author on reasonable request.

## Code availability

The source code used for this research work is made publicly available in the GitHub repository https://github.com/hds-69/csc-app.
