# Supplementary material for: Segmentation of lung lobes and lesions in chest CT for the classification of COVID-19 severity
Source: Sci Rep. 2023 Nov 28;13:20899. doi: 10.1038/s41598-023-47743-z (PMC10684885; doi:10.1038/s41598-023-47743-z)
Supplement: Supplementary file 1 — Supplementary Information. [file 41598_2023_47743_MOESM1_ESM.docx]

**Segmentation of Lung Lobes and Lesions in CT scans for Severity Classification of COVID-19**

Prachaya Khomduean^1^, Pongpat Phuaudomcharoen^2^, Totsaporn Boonchu^2^, Unchalisa Taetragool^3^, Kamonwan Chamchoy^4^, Nat Wimolsiri^2^, Tanadul Jarrusrojwuttikul^5^, Ammarut Chuajak^5^, Udomchai Techavipoo^6^, Numfon Tweeatsani^6,*^

^1^ Chulabhorn Learning and Research Centre, Chulabhorn Royal Academy, Bangkok, Thailand

^2^ Chulabhorn Hospital, Chulabhorn Royal Academy, Bangkok, Thailand

^3^ Department of Computer Engineering, Faculty of Engineering, King Mongkut’s University of Technology Thonburi, Bangkok, Thailand

^4^ Princess Srisavangavadhana College of Medicine, Chulabhorn Royal Academy, Bangkok, Thailand

^5^ Queen Savang Vadhana Memorial Hospital, Chonburi, Thailand

^6^ Faculty of Health Science Technology, HRH Princess Chulabhorn College of Medical Science, Chulabhorn Royal Academy, Bangkok, Thailand

* Corresponding author: Numfon Tweeatsani ([numfon.twe@cra.ac.th](mailto:numfon.twe@cra.ac.th))

**Supplementary Information**

**Abbreviations**

IoU Intersection over Union

DSC Dice Similarity Coefficient

HD Hausdorff Distance

CLAHE Contrast Limited Adaptive Histogram Equalization

RUL Right Upper Lobe

RML Right Middle Lobe

RLL Right Lower Lobe

LUL Left Upper Lobe

LLL Left Lower Lobe

**Supplementary Tables**

**Table S1.** Results of lung lobe model tests utilizing raw image sources from the Test Set 1 dataset.

| **Model** | **Backbone** | **IoU** | **DSC** | **Accuracy** | **Precision** | **Sensitivity** | **Specificity** | **HD (pixels)** |
| --- | --- | --- | --- | --- | --- | --- | --- | --- |
| 3D-UNet | DenseNet121 | 87.77 | 90.74 | 98.23 | 93.12 | 93.78 | 98.26 | 13.50 |
| **3D-UNet** | **DenseNet169** | **88.59** | **91.52** | **98.25** | **93.33** | **94.46** | **98.28** | **12.90** |
| 3D-UNet | DenseNet201 | 88.12 | 91.09 | 98.24 | 93.39 | 93.97 | 98.28 | 15.05 |
| 3D-UNet | ResNet18 | 85.26 | 88.72 | 98.19 | 92.33 | 91.91 | 98.29 | 16.66 |
| 3D-UNet | ResNet34 | 85.69 | 89.09 | 98.21 | 92.86 | 91.84 | 98.30 | 16.45 |
| 3D-UNet | ResNet50 | 87.36 | 90.39 | 98.22 | 92.42 | 94.12 | 98.25 | 14.85 |
| 3D-UNet | ResNet101 | 86.71 | 89.77 | 98.24 | 91.78 | 94.07 | 98.27 | 13.83 |
| 3D-UNet | ResNet152 | 86.69 | 89.95 | 98.21 | 92.37 | 93.38 | 98.26 | 15.33 |

**Table S2**. Lung lobe model test results using an image from the Test Set 1 dataset utilizing CLAHE technique.

| **Model** | **Backbone** | **IoU** | **DSC** | **Accuracy** | **Precision** | **Sensitivity** | **Specificity** | **HD (pixels)** |
| --- | --- | --- | --- | --- | --- | --- | --- | --- |
| 3D-UNet | DenseNet121 | 87.10 | 90.21 | 98.24 | 93.07 | 93.15 | 98.29 | 14.61 |
| **3D-UNet** | **DenseNet169** | **87.86** | **90.93** | **98.24** | **94.11** | **92.88** | **98.30** | **13.74** |
| 3D-UNet | DenseNet201 | 87.03 | 90.15 | 98.23 | 93.51 | 92.61 | 98.29 | 14.87 |
| 3D-UNet | ResNet18 | 85.08 | 88.43 | 98.19 | 91.54 | 92.55 | 98.27 | 16.12 |
| 3D-UNet | ResNet34 | 86.17 | 89.35 | 98.21 | 92.21 | 92.98 | 98.27 | 14.59 |
| 3D-UNet | ResNet50 | 86.59 | 89.85 | 98.22 | 92.40 | 93.24 | 98.27 | 15.12 |
| 3D-UNet | ResNet101 | 86.35 | 89.68 | 98.22 | 93.77 | 91.57 | 98.31 | 15.86 |
| 3D-UNet | ResNet152 | 86.14 | 89.37 | 98.23 | 93.35 | 91.80 | 98.30 | 15.89 |

**Table S3**. 3D-UNet + DenseNet169 testing result with original CT images of test set 1.

| **Case Type** | **DSC (%)** | | | | |
| --- | --- | --- | --- | --- | --- |
|  | **RUL** | **RML** | **RLL** | **LUL** | **LLL** |
| No lesion | 87.80 | 90.84 | 93.09 | 93.30 | 94.26 |
| Mild | 89.27 | 88.30 | 89.65 | 91.73 | 92.87 |
| Moderate | 92.94 | 92.06 | 93.16 | 91.55 | 93.82 |
| Severe | 88.05 | 91.02 | 89.26 | 94.30 | 93.18 |
| **Overall** | **89.52** | **90.56** | **91.29** | **92.72** | **93.53** |

**Table S4**. Lesion model test results using raw image source from Test Set 1 dataset.

| **Model** | **Backbone** | **IoU** | **DSC** | **Accuracy** | **Precision** | **Sensitivity** | **Specificity** | **HD (pixels)** |
| --- | --- | --- | --- | --- | --- | --- | --- | --- |
| 3D-UNet | DenseNet121 | 70.12 | 74.50 | 98.78 | 79.16 | 83.97 | 98.91 | 40.86 |
| **3D-UNet** | **DenseNet169** | **70.46** | **75.47** | **98.59** | **75.53** | **89.73** | **98.59** | **38.80** |
| 3D-UNet | DenseNet201 | 69.87 | 74.41 | 98.74 | 75.78 | 88.95 | 98.77 | 38.90 |
| 3D-UNet | ResNet18 | 60.66 | 61.09 | 99.24 | 85.12 | 62.55 | 99.98 | 60.00 |
| 3D-UNet | ResNet34 | 61.79 | 61.80 | 99.25 | 98.89 | 62.01 | 100 | 72.38 |
| 3D-UNet | ResNet50 | 61.92 | 62.04 | 99.24 | 91.33 | 62.13 | 100 | 72.90 |
| 3D-UNet | ResNet101 | 66.10 | 70.28 | 99.12 | 83.05 | 72.00 | 99.59 | 36.60 |
| 3D-UNet | ResNet152 | 60.41 | 62.54 | 99.18 | 80.99 | 64.51 | 100 | 40.09 |

**Table S5**. Lesion model test results with the image from the Test Set 1 dataset that applied the CLAHE technique.

| **Model** | **Backbone** | **IoU** | **DSC** | **Accuracy** | **Precision** | **Sensitivity** | **Specificity** | **HD (pixels)** |
| --- | --- | --- | --- | --- | --- | --- | --- | --- |
| 3D-UNet | DenseNet121 | 66.84 | 71.51 | 98.91 | 79.24 | 77.16 | 99.33 | 39.52 |
| **3D-UNet** | **DenseNet169** | **72.22** | **76.88** | **99.06** | **81.89** | **83.47** | **99.20** | **37.82** |
| 3D-UNet | DenseNet201 | 62.25 | 62.83 | 99.24 | 89.46 | 62.69 | 99.98 | 54.85 |
| 3D-UNet | ResNet18 | 61.03 | 61.36 | 99.25 | 87.60 | 62.37 | 99.99 | 55.34 |
| 3D-UNet | ResNet34 | 66.72 | 71.55 | 99.09 | 79.90 | 76.19 | 99.49 | 39.56 |
| 3D-UNet | ResNet50 | 61.98 | 62.00 | 99.25 | 99.22 | 62.02 | 100 | 68.06 |
| 3D-UNet | ResNet101 | 65.98 | 70.58 | 99.05 | 79.46 | 75.59 | 99.49 | 38.71 |
| 3D-UNet | ResNet152 | 67.99 | 71.86 | 99.16 | 85.83 | 73.59 | 99.55 | 42.58 |

**
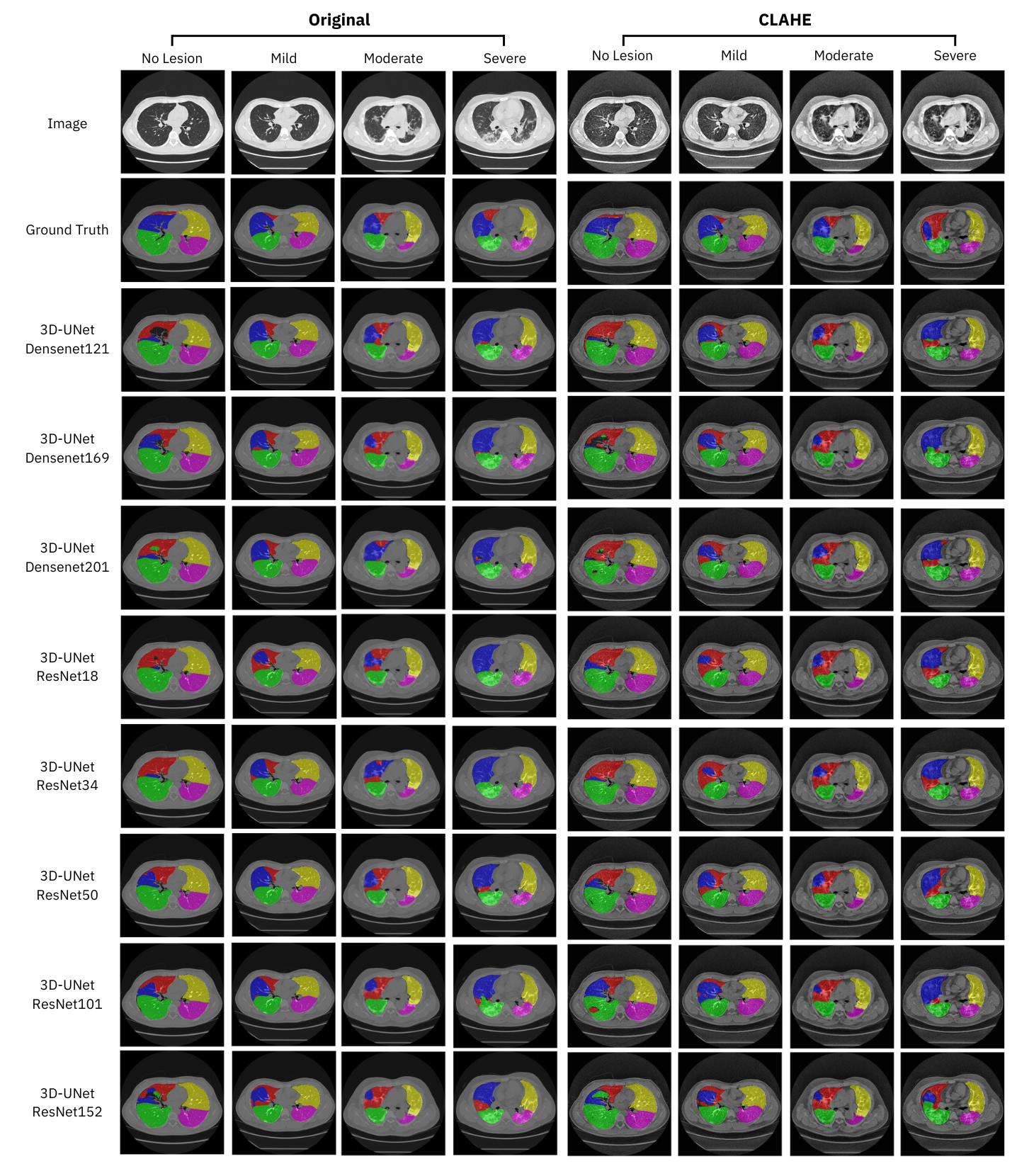
Supplementary figure**

**Figure S1.** Lung lobe segmentation results derived by the model trained on the original image and the CLAHE method. RUL, RML, RLL, LUL, and LLL refer to the colors Red, Blue, Green, Yellow, and Pink, respectively, for a detailed description of the colors representing each lobe of the lung.

**
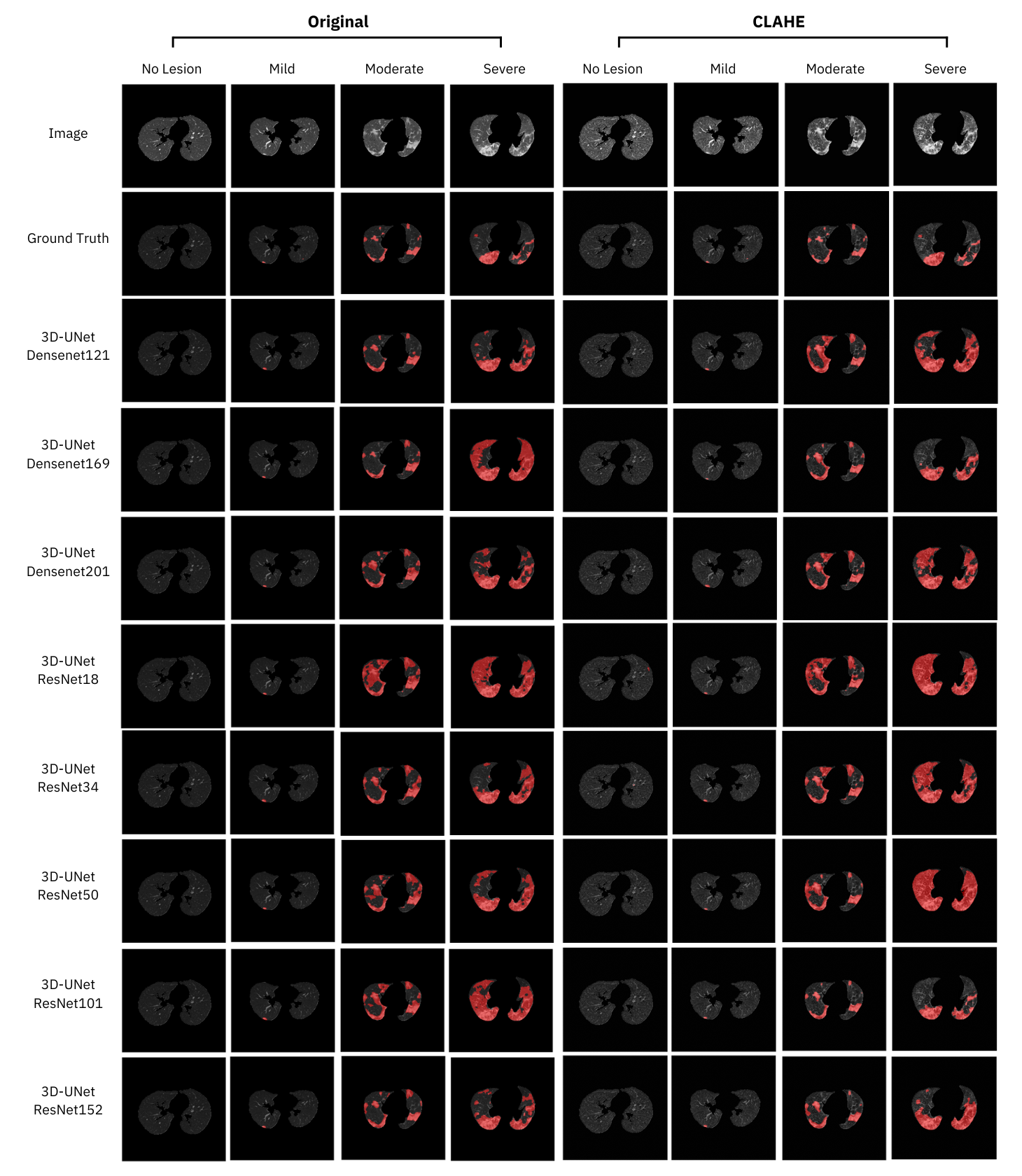
Figure S2.** Lesion segmentation results derived by the model trained on the original image and the CLAHE method. for a detailed description of the colors representing the lesion.
